# Identification and Precise Mapping of Resistant QTLs of Cercospora Leaf Spot Resistance in Sugar Beet (*Beta vulgaris* L.)

**DOI:** 10.1534/g3.111.000513

**Published:** 2011-09-01

**Authors:** Kazunori Taguchi, Tomohiko Kubo, Hiroyuki Takahashi, Hideyuki Abe

**Affiliations:** * National Agriculture and Food Research Organization (NARO), Hokkaido Agricultural Research Center (HARC), Memuro Upland Farming Research Division, Memuro, Hokkaido 082-0081, Japan and; † Laboratory of Genetics Engineering, Research Faculty of Agriculture, Hokkaido University, Sapporo, Hokkaido 060-8589, Japan

**Keywords:** Cercospora leaf spot, disease resistance, mapping, QTL, sugar beet

## Abstract

The complex inheritance of resistance to Cercospora leaf spot (CLS), the most severe fungal foliar disease in sugar beet, was investigated by means of quantitative trait loci (QTL) analysis. Over a three year period, recombinant inbred lines (RILs) of sugar beet (*Beta vulgaris* L.), generated through a cross between lines resistant (‘NK-310mm-O’) and susceptible (‘NK-184mm-O’) to CLS, were field-tested for their resistance to the pathogen. Composite interval mapping (CIM) showed four QTL involved in CLS resistance to be consistently detected. Two resistant QTL (*qcr1* on chromosome III, *qcr4* on chromosome IX) bearing ‘NK-310mm-O’ derived alleles promoted resistance. Across 11 investigations, the *qcr1* and *qcr4* QTL explained approximately 10% and over 20%, respectively, of the variance in the resistance index. Two further QTL (*qcr2* on chromosome IV, *qcr3* on chromosome VI) bearing ‘NK-184mm-O’ derived alleles each explained about 10% of the variance. To identify the monogenic effect of the resistance, two QTL derived from ‘NK-310mm-O’ against the genetic background of ‘NK-184mm-O’, using molecular markers. The *qcr1* and *qcr4* were precisely mapped as single QTL, using progenies BC_5_F_1_ and BC_2_F_1_, respectively. The *qcr1* that was located near e11m36-8 had CLS disease severity indices (DSI) about 15% lower than plants homozygous for the ‘NK-184mm-O’ genotype. As with *qcr1*, heterozygosis of the *qcr4* that was located near e17m47-81 reduced DSI by about 45% compared to homozygosis. These two resistant QTL might be of particular value in marker-assisted selection (MAS) programs in CLS resistance progression.

Caused by the fungus *Cercospora beticola* Sacc., Cercospora leaf spot (CLS), one of the most serious and widespread foliar diseases of sugar beet, typically provokes necrotic lesions, leading to a rapid and progressive destruction of the plant’s foliar apparatus (Holtschulte 2000). The continued replacement of new leaves occurs at the expense of reserve substances stored in sink tissues, and leads to a reduction in yield and sugar content. Yield losses of as much as 42 to 50% have been reported for CLS-infected beet crops (Smith and Martin 1978; Verreet *et al.* 1996). CLS control programs have sought to prevent disease infection by using resistant cultivars, applying fungicides, and rotating beets with non-host crops. As part of these efforts, sugar beet (*Beta vulgaris* L.) geneticists and breeders have sought to breed CLS resistance.

Lewellen and Whiteney (1976) identified monogenic resistance to the *C. beticola* race C2-induced CLS in a sugar beet cultivar; however, this resistance proved to be unstable and the cultivar was abandoned (Koch and Jung 2000). Some wild relatives of sugar beet have shown CLS resistance; *B. procumbens* C. Sm. shows CLS resistance, but is sexually incompatible with *B. vulgaris* (Panella and Frese 2000). However, some *B. vulgaris* spp. *maritima* accessions showing strong resistance to CLS have served as a source of CLS resistance in sugar beet (Leuterbach *et al.* 2004). An accession of *B. vulgaris* spp. *maritima* collected in the Po River delta by Dr. Munerati was backcrossed with sugar beets, and their resultant offspring became breeding material (Coons *et al.* 1955). These offspring reached the United States, were propagated there, and were then redistributed to the world (Skaracis and Biancardi 2000). The resistance achieved was effective in lowering the rate of infection in sugar beet, or in delaying the infection process (Rossi *et al.* 1996). However, the introduction of these resistance traits to other breeding lines was difficult given that their inheritance did not follow a simple Mendelian pattern, but rather was quantitative (Saito 1966). The resultant resistance was assumed to be controlled by at least four or five genes whose effects varied depending on the severity of infection (Smith and Gaskill 1970). Broad-sense heritability and realized heritability were estimated to be 60–70% and 25%, respectively, while variation caused by environmental factors ranged from 44 to 62% (Smith and Ruppel 1974). Due largely to environmental factors that affect the expression of resistance at the field level, mass selection for resistant phenotypes, either by natural infection or artificial inoculation, has made little progress. An apparent negative correlation between the CLS resistance and sugar yield (Saito 1966; Koch 1970) further complicated the task of these breeding programs. Genetic approach of CLS resistance, which can be aided by molecular markers, can help break the potential linkage between CLS resistance and unfavorable traits. Schäfer-Pregl *et al.* (1999) and Nilsson *et al.* (1999) conducted quantitative trait loci (QTL) analyses of CLS intensity in sugar beet lines consisting of an F_2_ population and F_3_ families. They reached a similar conclusion to Smith and Gaskill (1970), namely that at least four or five QTL were involved. Similarly, two QTL analyses conducted by Setiawan *et al.* (2000)—one based on a field test under natural infection condition and another using a leaf disk test—detected at least four QTL. While these studies were pivotal in elucidating the genetics of CLS resistance in sugar beet, questions remained as to the precise map positions of the QTL, as well as their respective gene products and effects.

We launched a multitask research program on the genetic analysis of sugar beet resistance to multiple diseases, including Aphanomyces root rot, CLS and Rhizoctonia root rot. An initial field-based screening of Japanese sugar beet lines identified breeding line ‘NK-310mm-O’ as a source of a high level of resistance to multiple diseases (Taguchi *et al.* 2007). This prompted us to characterize the genetic nature of these resistance traits, with a goal of establishing a marker-assisted selection (MAS) system for resistance to these diseases. This would enable the rapid development of a sugar beet line with all known resistances within a short time. As a part of this program, we focused our analysis on the genetics of resistance to CLS. The objectives of this study were to: (1) identify QTL for CLS resistance within our breeding material under field conditions, (2) resolve the QTL into individual genetic factors using recombinant inbred lines (RILs), and (3) evaluate the genetic effect of each QTL using near isogenic lines (NILs) produced by recurrent back crosses.

## Materials and Methods

### Plant material

Sugar beet lines used in this study were developed by the National Agricultural and Food Research Organization (NARO) at the Hokkaido Agricultural Research Center (HARC), Japan. A resistant line ‘NK-310mm-O’ and a susceptible line ‘NK-184mm-O’ were O-type, in which all the genes for fertility restoration of Owen-type cytoplasmic male sterility (CMS) were homozygous for non-restoring alleles. An F_1_ plant was obtained from the cross between ♀ ‘NK-310mm-O’ and ♂ ‘NK-184mm-O’, then self-pollinated to generate F_2_ seeds. To produce F_3_ lines, each of one specimen parental plant was physiologically isolated to prevent cross-pollination. 80 RILs were created in the F_6_ to F_7_ generation, alternating with a single-seed descent method after F_3_ lines. ‘NK-184mm-CMS’ was an isoplasmic line whose nuclear genotype was almost identical to ‘NK-184mm-O’ but which possessed Owen CMS. The five sets of NILs and BLs (BC_3_F_3_ and BC_4_F_1_-CMS) were already constructed at HARC by a recurrent backcross scheme (Taguchi *et al.* 2010). The segregation population of BLs (BC_5_F_1_-CMS & BC_2_F_1_-CMS) was prepared by crossing ‘NK-184mm-O’ to each single plant with backcrossed progeny. ‘Monohomare’, ‘Monohikari’, ‘Yukihinode’ and ‘Stout’ were commercial sugar beet cultivars.

### Inoculation with *Cercospora beticola* and resistance evaluation

Field trials were carried out in HARC fields, in Memuro, Japan. For initial QTL analysis, the experiment was set up as a randomized block with four replications (2005 and 2006) or two replications (2007). Individual plot size was 1.35 m^2^, and the final plant density was ten plants per plot (= 70000 plants ha^−1^). For selection of backcross progenies, a similar design, with four replications, was used in 2008. For precise QTL mapping, (conducted in 2009), sugar beets were planted in a zigzag pattern; each subject plant was enclosed by a barrier of resistant plants, to prevent them from contacting each other (Taguchi *et al.* 2002). The initial plant density was 70,000 plants ha^−1^, and declined to roughly 3500 plants ha^−1^ after the removal of barrier plants, before the evaluation of CLS resistance. Individual plot size was 32.4 m^2^, and the final plant density was 120 plants.

Seeds were sown in paper pots (19 mm diameter and 13 cm height, Nippon Beet Sugar Mfg. Co., Ltd.) in early April. One month later, seedlings were transplanted to the field. *Cercospora beticola* inoculum was prepared as follows: petioles of sugar beet leaves expressing severe CLS symptom were collected from HARC fields, dried, and ground to a powder. In early July of the subsequent year, inoculum (5 g) was applied at the foot of each plant. Initial symptoms were observed roughly one month after inoculation. Visual symptoms of CLS were rated on an index ranging from zero for no symptoms, to five for fully destroyed main leaves in each 10 plants per replication. Data for RILs, NILs and BLs were averaged across replications in each investigation time.

### DNA isolation and genotyping with molecular markers

Total cellular DNA was extracted from fresh leaves according to the procedure of Roger and Bendich (1988). Amplified Fragment Length Polymorphism (AFLP) was detected using an AFLP Analysis System I (Invitrogen, Carlsbad, CA, USA). The restriction endonucleases *Eco*RI and *Mse*I were used in this analysis. The adapter-ligated DNA was pre-amplified with primers having a single selective nucleotide. For selective amplification, *Eco*RI-NNN and *Mse*I-NNN primers were employed using primer sets (Taguchi *et al.*, 2009). The amplified products were electrophoresed using a High Efficiency Genome Scanning (HEGS) system (Hori *et al.* 2003; Kikuchi *et al.* 2003), in which discontinuous non-denatured polyacrylamide gel and TBE buffer were used. The gels were scanned after staining with Vistra Green I (GE Healthcare UK, Amersham Place, England) or Sybr green I (Molecular Probes, Eugene, OR, USA) and photographed under a UV transilluminator (ATTO, Tokyo, Japan). Cleaved Amplified Polymorphic Sequence (CAPS) markers were developed as follows. PCR products were generated using primer sets, as described by Möhring *et al.* (2005), Hunger *et al.* (2003), and Schneider *et al.* (1999), then digested with one of thirteen restriction endonucleases: *Hae*III, *Hha*I, *Taq*I, HapII, *Mbo*I, AfaI, XspI, AluI, and AccII (Takara Bio, Ohtsu, Japan); TspEI (TOYOBO, Osaka, Japan); and *Mse*I, HpyCH4IV, and NlaIII (New England BioLabs, Beverly, MA, USA). The resultant fragments were electrophoresed in 2% agarose gels to check for polymorphism. Other PCR markers including simple sequence repeat (SSR) markers were based on McGrath *et al.* (2007), Viard *et al.* (2002), Laurent *et al.* (2007), and Hagihara *et al.* (2005). The cycling parameters were 40 cycles of 94°C for 1 min and 50–60°C (depending on the primers) for 1 min, followed by one cycle at 72°C for 10 min. The amplified products were electrophoresed using the HEGS system. To identify CLS resistance QTL in ‘NK-310mm-O,’ a linkage map was constructed using the 80 RILs, using DNA markers such as AFLP, CAPS, and SSRs. Seventy-nine selected AFLP markers, whose map positions had been examined using an F_2_ population derived from a cross between ‘NK-310mm-O’ and ‘NK-184mm-O’ (Taguchi *et al.* 2009), were deemed to effectively cover the nine linkage groups of sugar beet. For CAPS markers, PCR primers were prepared based on SNP marker sets (Möhring *et al.* 2005) and STS from resistant gene analogs (RGAs) (Hunger *et al.* 2003). A total of 1287 combinations (99 primer sets and 13 restriction endonucleases) were tested in an effort to detect polymorphism between the parental lines. Of 78 primer combinations, 10 were polymorphic between the parental lines. MP-A16 was a PCR marker which co-segregated with *X*, a nuclear fertility restorer for Owen CMS (Hagihara *et al.* 2005).

### Linkage map construction and QTL mapping

Polymorphic bands among the RILs were checked by their coupling to ‘NK-310mm-O’, as well as segregation. The multiple segregation data were manually scored using a Microsoft Excel spreadsheet (Microsoft Japan, Tokyo, Japan). Segregation data of AFLP, SSR, and CAPS markers were grouped at a logarithm of odds (LOD) threshold of 3.0 and a maximum distance of 25 cM. Marker order in each of the linkage groups was verified by using MAPMAKER/EXP version 3.0 (Lander *et al.* 1987). The Kosambi mapping function was used to calculate the map distance (Kosambi 1944). QTL analysis was carried out by one of two methods: (1) composite interval mapping (CIM) using Win QTL Cartographer version 2.5 (Wang *et al.* 2007) using a permutation test with 1000 permutations and a mean LOD threshold sufficient to declare a putative QTL as being significant or not, or (2) simple interval mapping (SIM) methods with MAPMAKER/QTL1.1 (Lincoln *et al.* 1993).

## Results

### Evaluation of the Cercospora leaf spot resistance

To genetically characterize the CLS resistance possessed by ‘NK-310mm-O’ and progeny generated. Based on plant symptoms expressed after infection promoted by inoculums, CLS resistance was assessed in field trials. ‘NK-310mm-O’ scored the lowest or second lowest disease severity index (DSI) in every year (Table 1), indicating its strong resistance to CLS. On the other hand, 'NK-184mm-O’ scored the highest or the second highest DSI, and was considered highly susceptible. An ANOVA between parental lines indicated that ‘NK-310mm-O’ was significantly more resistant to CLS than ‘NK-184mm-O’ (Table 1). In 2006 and 2007, the field trials included three commercial sugar beet varieties, ‘Monohomare’, ‘Monohikari’ and ‘Yukihinode,’ whose CLS resistance was known to be medium-weak, medium, and strong, respectively (Taguchi *et al.* 2007). In both years, their DSIs consistently followed the order of their CLS resistances (Table 1), thus validating the field trials. The DSIs in 2007 were higher than those in 2005, but comparable to those of 2006.

**Table 1:** Disease severity indices (DSI) means for Cercospora leaf spot (CLS) under inoculated field evaluation

	2005			2006				2007			
Varieties	9.05	9.12	9.20	8.08	8.17	8.23	8.28	8.13	8.20	8.28	9.02
Monohomare (medium-weak)	—	—	—	1.3	3.7	4.4	4.7	2.3	3.5	4.3	4.9
Monohikari (medium)	—	—	—	1.0	3.1	4.0	4.1	1.7	2.6	3.4	4.2
Yukihinode (strong)	2.5	3.1	4.1	0.4	2.5	3.4	3.5	0.4	1.6	2.6	3.4
NK-310mm-0 (very strong)	1.3	1.5	2.1	0.2	1.7	2.7	2.9	0.4	1.9	2.6	3.3
NK-184mm-0 (weak)	4.3	4.6	4.9	1.2	4.0	4.9	5.0	2.9	3.9	4.9	5.0
F1	2.0	2.6	3.0	1.0	2.8	3.5	3.9	1.0	2.8	3.3	3.8
F-test	**	**	**	**	**	**	**	**	**	**	**
LSD(5%)	0.7	0.7	0.6	0.3	0.5	0.4	0.3	0.4	0.5	0.6	0.5
LSD(1%)	1.0	0.9	0.8	0.4	0.6	0.5	0.4	0.5	0.7	0.8	0.6

^**^p < 0.01. LSD, least significant differences.

The DSI of F_1_ plants derived from the ‘NK-310mm-O’ × ‘NK-184mm-O’ cross were close to the mean of their parent values. Therefore, the CLS resistance appeared to behave in an additive manner. We developed 80 RILs from a single F_1_ plant. Their resistance to CLS was examined in the field trials (2005, 2006 and 2007). Table 2 shows the frequency distribution of DSIs among the 80 RILs. Year-to-year correlations of medium to late season DSI assessments showed R^2^ values of 0.59, 0.69, and 0.55 for 2005/2006, 2006/2007, 2005/2007 comparisons, respectively. These figures indicate that the CLS resistance was expressed in a stable manner over the three years.

**Table 2:** Frequency distribution for CLS indices in RILs of ‘NK-310mm-O’ × ‘NK-184mm-O’

			Mean DSI		Frequency of DSI for Cercospora Leaf Spot					
Year	Date	Intensity	Mean	± SEM	0	1	2	3	4	5
2005 (*n* = 80)	9.05	medium-early	2.3	1.1	0	15	17	29	15	4
	9.12	medium-late	3.0	1.1	0	3	17	21	22	17
	9.02	late	4.0	1.0	0	0	4	13	18	45
2006 (*n* = 80)	8.08	early	0.7	0.3	0	68	12	0	0	0
	8.17	medium-early	2.8	0.7	0	0	14	31	33	2
	8.23	medium-late	3.8	0.6	0	0	0	8	42	30
	8.28	late	4.1	0.6	0	0	0	4	28	48
2007 (*n* = 80)	8.13	early	1.3	0.9	0	43	21	15	1	0
	8.20	medium-early	2.4	1.1	0	16	19	31	11	3
	8.28	medium-late	3.3	1.1	0	5	9	19	32	15
	9.02	late	3.9	1.0	0	0	8	9	22	41

DSI, disease severity index (0–5 scale [0 = no symptoms, 5 = almost complete necrosis]); SEM, standard error of the mean.

### Construction of genetic framework for QTL analysis

**Figure 1:**
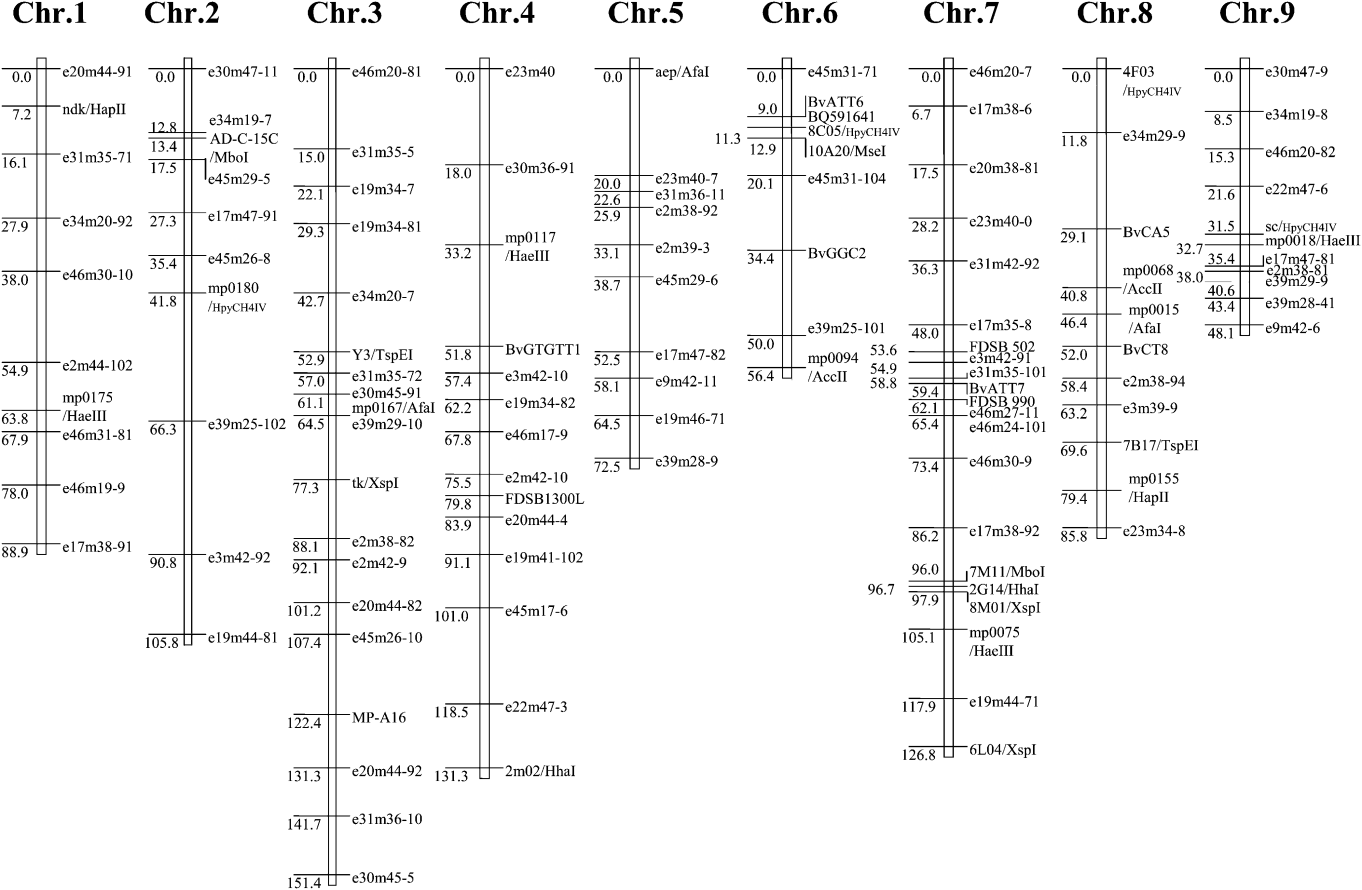
Linkage map based on RILs ('NK-310mm-O' ×'NK-184mm-O'). Markers labeled “e^**^m^**^-^**^” were AFLP markers, while markers labeled “^****^/^****^” were CAPS markers. Marker intervals are indicated in cM. The total map length is 867 cM.

Altogether, the resultant map covered 867cM, including the 79 AFLP, 25 CAPS, 10 SSR, and 1 PCR markers, with a mean distance between loci of 7.5 cM (Figure 1), among which nine linkage groups were apparent. The assignment of the nine linkage groups to the nine sugar beet chromosomes (Butterfass 1964; Schondelmaier and Jung 1997) was succeeded by anchoring the CAPS markers and the MP-A16. The order of some of the AFLP markers was slightly different from the previous linkage map described by Taguchi *et al.* (2009), but the grouping of the markers was not changed.

### QTL analysis for Cercospora leaf spot resistance

Eleven sets of the 80 RILs DSI (Table 2) were analyzed by a CIM method in order to identify the relevant QTL (Table 3). In each of the data sets, two to five significant LOD peaks were detected on chromosomes II, III, IV, VI, and IX. The LOD peaks on chromosomes III, IV, VI, and IX were detected from more than eight data sets. Moreover, the location of the LOD peaks was largely consistent, indicating the presence of QTL for CLS resistance. The QTL on chromosomes III, IV, VI, and IX were named *qcr1*, *qcr2*, *qcr3*, and *qcr4*, respectively (supporting information, Figure S1).

**Table 3:** QTL associated with resistance to CLS identified in the RILs of ‘NK-310mm-O’ × ‘NK-184mm-O’ by the CIM method

Year	Date	Chr.	LOD^*a*^	Threshold	Significant Marker Region*^b^*			R^2^ *^c^*	Total R^2^	Additive
2005	9.05	III	3.17	2.8	e20m44-82	∼	e20m44-92	11%		−0.40
	(ME)	VI	5.52	2.8	e45m31-71	∼	8C05/HpyCH4IV	16%		0.44
		IX	8.85	2.8	e22m47-6	∼	e39m28-41	27%	54%	−0.58
	9.12	III	3.28	2.8	e20m44-82	∼	e20m44-92	13%		−0.45
	(ML)	IV	3.01	2.8	e30m36-91	∼	mpll7/*Hae*III	10%		0.35
		VI	3.65	2.8	e45m31-71	∼	8C05/HpyCH4IV	12%		0.40
		IX	8.59	2.8	e46m20-82	∼	e39m28-41	28%	63%	−0.64
	9.20	VI	3.85	2.8	e45m31-71	∼	8C05/HpyCH4IV	13%		0.35
	(L)	IX	5.57	2.8	e22m47-6	∼	e39m28-41	20%	33%	−0.44
2006	8.08	II	2.80	2.7	e30m47-ll	∼	e34ml9-7	9%		0.11
	(E)	III	3.13	2.7	tk/XspI	∼	e45m26-10	12%		−0.12
		IV	3.47	2.7	e46ml7-9	∼	e20m44-4	11%		0.11
		VI	4.62	2.7	e45m31-71	∼	e45m31-104	16%		0.14
		IX	6.18	2.7	e22m47-6	∼	e9m42-6	21%	69%	−0.17
	8.17	III	3.18	2.8	tk/XspI	∼	e2m38-82	8%		−0.20
	(ME)	IV	2.86	2.8	FDSB 1300L	∼	e20m44-4	8%		0.19
		VI	3.21	2.8	BvGGC2	∼	mp94/AccII	10%		0.23
		IX	10.95	2.8	e22m47-6	∼	e39m28-41	34%	60%	−0.44
	8.23	IV	4.22	2.6	e46ml7-9	∼	e20m44-4	13%		0.21
	(ML)	VI	4.47	2.6	e45m31-71	∼	e45m31-104	13%		0.22
		IX	9.29	2.6	e22m47-6	∼	e9m42-6	30%	56%	−0.35
	8.28	III	3.58	2.7	tk/XspI	∼	e2m42-9	13%		−0.21
	(L)	IX	5.33	2.7	e22m47-6	∼	e39m28-41	20%	33%	−0.26
2007	8.13	III	3.11	2.7	tk/XspI	∼	e45m26-10	11%		−0.32
	(E)	IV	4.94	2.7	mpll7/*Hae*III	∼	el9m34-82	17%		0.57
		IV	3.64	2.7	2M02/*Hha*I	∼	e22m47-3	14%		0.41
		IX	3.62	2.7	e22m47-6	∼	el7m47-81	12%	54%	−0.27
	8.20	III	2.79	2.7	tk/XspI	∼	e2m38-82	8%		−0.32
	(ME)	IV	4.08	2.7	mpll7/*Hae*III	∼	el9m34-82	13%		0.42
		VI	3.16	2.7	e45m31-71	∼	8C05/HpyCH4IV	9%		0.32
		IX	6.88	2.7	e22m47-6	∼	e39m28-41	20%	50%	−0.48
	8.28	III	2.80	2.8	tk/XspI	∼	e2m38-82	9%		−0.35
	(ML)	IV	3.13	2.8	mpll7/*Hae*III	∼	BvGTGTTl	12%		0.37
		IX	8.60	2.8	e22m47-6	∼	e39m28-41	29%	50%	−0.62
	9.02	IV	3.88	2.8	mp117/*Hae*III	∼	e3m42-10	15%		0.40
	(L)	VI	3.25	2.8	e45m31-71	∼	8C05/HpyCH41V	10%		0.32
		IX	6.85	2.8	e22m47-6	∼	e39m28-41	25%	50%	−0.50

a Log of the odd probability of detecting a QTL in a particular place.

b Position of the significant LOD peak of the QTL in relation to the first marker of given interval.

c Percentage of explainable variation.

The actions of the four QTL were contrasting: *qcr1* and *qcr4* decreased DSI when plants had ‘NK-310mm-O’ alleles, and their effects were additive: −0.12 to −0.45 for *qcr1* and −0.17 to −0.64 for *qcr4*. The CLS resistance conferred by *qcr4* was always greater than that derived from *qcr1* (Table 3). QTL *qcr2* and the *qcr3* were also additive, but increased DSI in plants bearing ‘NK-310mm-O’ alleles. The additive effects of the *qcr2* and the *qcr3* were estimated to be 0.11 to 0.57, and 0.14 to 0.44, respectively. The ‘NK-310mm-O’ genotypes with respect to the marker mp0117/*Hae*III around *qcr2* had DSI that was an average of 14% higher than the ‘NK-184mm-O’ genotype in RILs. Around the *qcr3 BvATT6*, it had DSI that was an average of 18% higher than the ‘NK-184mm-O’ genotypes.

### Verifying the allelic differences on the detected resistance QTL

**Figure 2:**
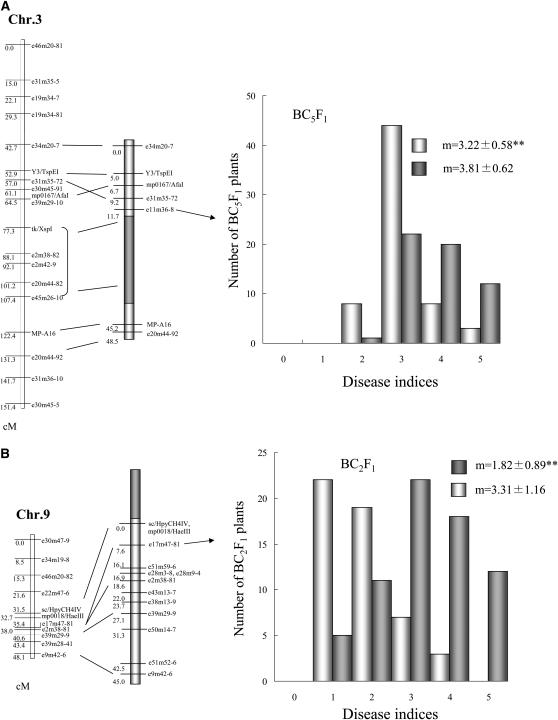
Precise linkage map around the detected two resistant QTL, *qcr1* (A) and *qcr4* (B). The frequency distribution for CLS indices in BC_5_F_1_ (A) and BC_2_F_1_ (B). Different genotypic classes, as defined at the nearest marker locus for the QTL peak in each population. White represents individuals heterozygous for the allele from the resistant parent, and gray represents individuals homozygous for allele from the susceptible parent. ^**^p < 0.01. m, mean ± SE.

In examining the phenotype of plants having either of the two resistant QTL, *qcr1* and *qcr4*, *qcr1*, it was discovered to be located near *Acr1*, a resistance gene for Aphanomyces root rot whose source was ‘NK-310mm-O’ (Taguchi *et al.* 2010). The hypothesis that map positions of *qcr1* and *Acr1* being so close meant that plants selected for *Acr1* were also CLS resistant due to the linked *qcr1* was tested. Five near isogenic lines (NILs; BC_3_F_3_) and five back-crossed lines (BLs; BC_4_F_1_) selected for a probable chromosomal region containing the *Acr1* QTL but having an otherwise ‘NK-184mm-O’ nuclear background (Taguchi *et al.* 2010) were planted to examine CLS resistance. With a mean DSI (Sept. 10) of 2.4, ‘NK-310mm-O’ plants exhibited a lower DSI than the strong resistant variety ‘Stout’ on (Table 4). Under the same conditions, ‘NK-184mm-O’ plants were severely damaged (mean DSI of 4.6). The DSIs of the five NILs and the five BLs-CMS lines ranged from 3.5 to 4.8, of which NIL-2, NIL-3, BL-CMS-2, and BL-CMS-3 were the most resistant (Table 4). One of the two resistant backcross lines, BL-CMS-2, which was absent from tk/XspI to e10m37-9 (Taguchi *et al.* 2010), was selected and crossed with ‘NK-184mm-O’ to generate a population segregating the CLS resistance likely governed by the *qcr1*. In 2009, QTL analysis of CLS resistance in this population (BC_5_F_1_) was conducted using a SIM method. The LOD score and the estimated additive effect assessed through the SIM method are presented in Table 5. The *qcr1* was located near the PCR marker e11m36-8, on chromosome III (Figure 2A). The explained variance was roughly 45% (Table 5). Plants heterozygous with respect to genotype marker around *qcr1* had Cercospora leaf spot disease severity indices (DSI) about 15% lower than plants homozygous for the ‘NK-184mm-O’ genotype. To assess the effect of *qcr4*, graphical genotypes of the 80 RILs derived from 115 molecular markers were used to select lines bearing the *qcr4* but not the *qcr1* QTL. Line ‘RIL 56’ was selected as meeting this criterion, and was crossed with ‘NK-184mm-CMS.’ The resultant B_1_F_1_ was back-crossed with ‘NK-184mm-O’, to generate two BC_2_F_1_-CMS populations each segregating the *qcr4*. In 2009, plants of the population were planted for QTL analysis of CLS resistance by the SIM method. The *qcr4* was located near the PCR marker e17m47-81, on chromosome IX (Figure 2B). The explained variance was roughly 46%. Plants heterozygous with respect to genotype marker around *qcr1* had Cercospora leaf spot disease severity indices (DSI) about 45% lower than plants homozygous for the ‘NK-184mm-O’ (Table 5).

**Table 4:** DSI means for NILs and BLs under evaluation in CLS-infected field trials

	DSI		
Line Name	Aug. 8	Aug. 25	Sept. 10
Monohomare	0.5	2.1	4.1
Monohikari	0.4	2.1	4.0
Stout	0.4	1.2	3.1
NK-184mm-O	0.6	2.6	4.6
NK-310mm-O	0.1	0.6	2.4
NIL-1	0.7	2.9	4.8
NIL-2	0.5	1.6 ^**^	3.5 ^**^
NIL-3	0.3	1.9 ^*^	4.0 ^**^
NIL-4	0.8	2.3	4.7
NIL-5	0.2	2.3	4.8
BL-CMS-1	0.6	2.6	4.8
BL-CMS-2	0.2	1.8 ^**^	3.9 ^**^
BL-CMS-3	0.5	2.1 ^*^	4.0 ^**^
BL-CMS-4	0.7	2.6	4.3
BL-CMS-5	0.3	2.0	4.6
Average	0.4	2.0	4.1
F-Test	*	**	**
LSD (5%)	0.4	0.7	0.4
LSD (1%)	0.5	0.9	0.5

Lines were compared to ‘NK-184mm-O’. ^**^p < 0.01, ^*^p < 0.05. LSD, least significant differences.

**Table 5:** A single QTL locus associated with resistance to CLS identified in the BLs of ‘NK-310mm-O’ × ‘NK-184mm-O’ by the SIM method

Material	Plant Number	Average of DSI	Chromosome	Significant Marker Region*^a^*			LOD*^b^*	R^2^ *^c^*	Additive
B_5_F_1_	120	3.49 ± 0.67	3	e34m20-8	∼	e20m44-92	6.1	44.8%	−0.97
B_2_F_1_	120	2.73 ± 1.22	9	sc/HpyCH4IV	∼	e9m42-6	15.7	45.8%	−1.75

a Position of the significant LOD peak of the QTL in relation to the first marker of given interval.

b Log of the odd probability of detecting a QTL in a particular place.

c Percentage of explainable variation.

## Discussion

Genetic analysis of CLS resistance in sugar beet, carried out in a number of previous studies, revealed the quantitatively inherited nature of the resistance (Smith and Ruppel 1974; Saito 1966), but the chromosomal location of the genes responsible remained obscure. This occurred because the CLS resistance introduced from wild relatives may have been constituted by multiple genes with weak effects, making it difficult to identify individual genes as Mendelian factors. To overcome this difficulty, genetic analysis of a resistant source using well-characterized genetic stocks, such as RILs and NILs, may be a solution. Consequently, in the present study we sought to identify QTL for CLS resistance from ‘NK-310mm-O’ under the field conditions.

The four QTL identified (*qcr1*, *qcr2*, *qcr3* and *qcr4*) affected CLS resistance differently. The *qcr4* QTL was the most stable of the four, showing large LOD scores in all eleven trials, and explained over 20% of phenotypic variance in all investigations. The other three QTL were rather unstable compared to the *qcr4*, their LOD peaks sometimes being beneath the threshold of significance. This might be attributable to environmental factors and/or plant conditions. For example, Saito (1966) pointed out that variation in CLS resistance was influenced by leaf age. Moreover, alleles of the *qcr2* and the *qcr3* of ‘NK-310mm-O’ appeared to confer CLS susceptibility, which led us to infer that, although ‘NK-310mm-O’ was the highly resistant line, there might be room to improve its resistance to CLS.

The *qcr1* and the *qcr4* were mapped to chromosomes III and IX, respectively. Various sugar beet chromosomes have been associated with QTL for CLS resistance; Schäfer-Pregl *et al.* (1999) detected LOD peaks on chromosomes II, III, VI, and IX in F_3_ families and on chromosomes IV and V in F_2_ data. Nilsson *et al.* (1999) mapped five QTL on chromosome I, II, III, and IX, of which two were on chromosome III (T. Kraft, personal communication). The QTL mapped by Setiawan *et al.* (2000) were on chromosomes IV, VII, VIII (two QTL), and IX in their field test, and III, IV, VII, and IX in their leaf disc test. Because their experimental conditions and genetic model differed from ours, a direct comparison of results may be inappropriate; however, it seems significant that chromosomes III and IX have always been associated with the QTL of CLS resistance. Thus, it appears possible that CLS resistance in sugar beet involves genes located on chromosomes III and IX.

For MAS selection or molecular investigation of the *qcr1* and *qcr4* QTL, knowledge regarding their individual effects and precise map position will be useful. We genetically approached the CLS resistance to clarify the contribution of the individual *qcr1* and *qcr4* QTL. A similar approach was used in dissecting rice heading QTL (Yano *et al.* 1997; Yamamoto *et al.* 2000). Assuming that molecular markers are a sufficiently stringent criterion for the introduction (as is now feasible) of a candidate chromosomal region of interest into progeny for verifying the detected QTL, particular care must be taken to accurately evaluate phenotypes, as the effect of a single QTL may be small. Therefore, individual subject plants were isolated from one another by planting CLS resistant beets as barriers. This procedure was expected to prevent subjects from touching infected beets. Subsequently, it caused easier observation of the whole plant phenotype. As a result, map positions of the *qcr1* and the *qcr4* were confined to chromosomal segments, and could be estimated as single QTL. Explained variance figures for the *qcr1* and *qcr4* QTL suggested that plants having one or both QTL in the heterozygous form exhibited higher resistance than ‘NK-184mm-O.’

The map position of the *qcr1* is consistent with the location of the resistance gene cluster in the sugar beet genome. A number of important sugar beet disease resistance genes have been mapped to chromosome III; CLS resistance QTL (Schäfer-Pregl *et al.* 1999; Setiawan *et al.* 2000; Nilsson *et al.* 1999), Rhizomania resistance genes *Rz1* to *Rz5* (Barzen *et al.* 1999; Scholten *et al.* 1999; Gidner *et al.* 2005; Grimmer *et al.* 2007), and Aphanomyces root rot resistance gene *Acr1* (Taguchi *et al.* 2010). In addition, a number of resistance gene analogs (RGAs) have been cloned from sugar beet, some of which have been mapped to a gene cluster on chromosome III (Lein *et al.* 2007). Further study of sugar beet genomics is forthcoming to clarify the evolution of sugar beet resistance gene clusters.

The chromosome III also contains another important gene, *X*, a restorer of fertility for Owen CMS (Owen 1945; Hagihara *et al.* 2005). The map position of the *X* is represented by a molecular marker MP-A16, a PCR marker which co-segregates with *X*. The close linkage of the *X*, *qcr1*, and *Acr1* suggests a possible linkage drag in the breeding of ‘NK-310mm-O’. This line, which originated from breeding line ‘Tmm-1’, a Japanese donor source of the monogerm trait, was conferred by the *m* gene on chromosome IV (Barzen *et al.* 1995;Schondelmaier and Jung 1997). Although it appeared to be a highly heterogeneous population, no one had recognized any disease resistance traits in ‘Tmm-1’. In the 1960s, ‘Tmm-1’ was subjected to the selection for maintainer genotypes to obtain a breeding line having both the monogerm trait and maintainer genotype. After the second-round maintainer selection, a pedigree whose Aphanomyces root rot resistance was as high as that of ‘NK-310mm-O’ emerged (data not shown). The maintainer selection increased the frequency of a non-restoring allele of *X*, known to be very rare in the sugar beet population (Bosemark 2006). Since *Acr1* and *qcr1* are linked to the non-restoring allele (*i.e.*, ‘NK-310mm-O’ is a maintainer line), the maintainer selection may have resulted in an increased frequency of *Acr1* and *qcr1* QTL, which likely lurked as infrequent alleles in ‘Tmm-1’. Although our speculation needs to be supported by additional data and other scenarios are possible, there may be concern that maintainer selections can cause an unintentional decrease of the genetic diversity in the resistance-gene cluster on the chromosome III, which is involved in some of the major disease resistances of sugar beet.

## Supplementary Material

Supporting Information
