# Supplementary material for: Identification and Precise Mapping of Resistant QTLs of Cercospora Leaf Spot Resistance in Sugar Beet (Beta vulgaris L.)
Source: G3 (Bethesda). 2011 Sep 1;1(4):283–91. doi: 10.1534/g3.111.000513 (PMC3276142; doi:10.1534/g3.111.000513)
Supplement: Supporting Information [file supp_1_4_283__index.html]

Supporting Information 

# Identification and Precise Mapping of Resistant QTLs of Cercospora Leaf Spot Resistance in Sugar Beet (*Beta vulgaris* L.)

## Supporting Information for Taguchi *et al.*, 2011

**Files in this Data Supplement:**

- Figure S1 - Putative QTLs for CLS detected in RILs by CIM method (PDF, 84 KB)
