# Supplementary material for: Identification and Precise Mapping of Resistant QTLs of Cercospora Leaf Spot Resistance in Sugar Beet (Beta vulgaris L.)
Source: G3 (Bethesda). 2011 Sep 1;1(4):283–91. doi: 10.1534/g3.111.000513 (PMC3276142; doi:10.1534/g3.111.000513)
Supplement: Supporting Information [file supp_1.4.283_FigureS1.pdf]

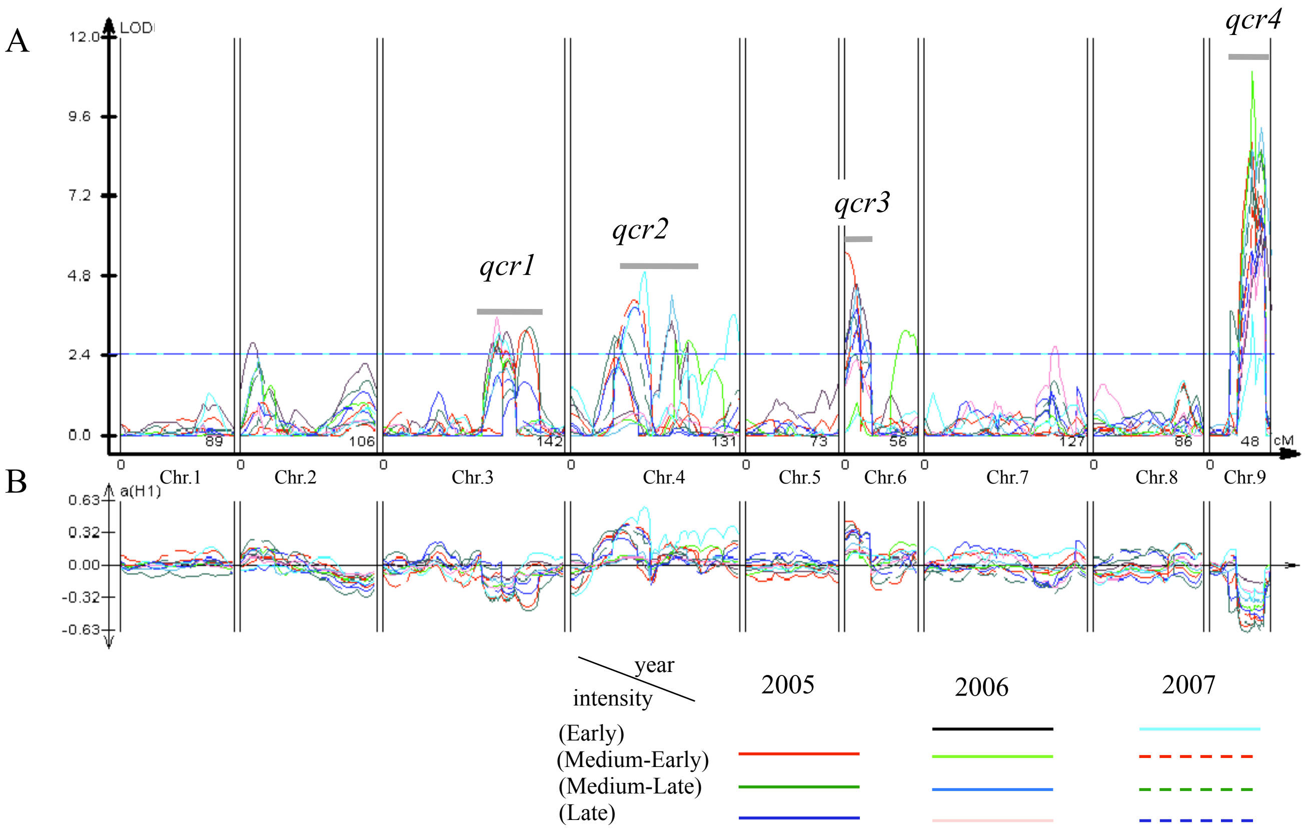

Figure S1 Putative QTLs for CLS detected in RILs by CIM method. Individual significant regions were frequently detected on chromosomes III, IV, VII and IX, named as *qcr1*, *qcr2*, *qcr3* and *qcr4*, respectively. A shows the variation in LOD score and B shows the variation in additive effect in each chromosome.
